# Quality of Life and Excessive Daytime Sleepiness in Adults with Obstructive Sleep Apnea Who Are Treated with Multilevel Surgery or Adherent to Continuous Positive Airway Pressure

**DOI:** 10.3390/jcm11092375

**Published:** 2022-04-23

**Authors:** Giannicola Iannella, Giuseppe Magliulo, Cristina Anna Maria Lo Iacono, Irene Claudia Visconti, Jerome R. Lechien, Tiziano Perrone, Giovanni Cammaroto, Giuseppe Meccariello, Antonino Maniaci, Salvatore Cocuzza, Milena Di Luca, Andrea De Vito, Chiara Martone, Antonella Polimeni, Antonio Greco, Marco de Vincentiis, Massimo Ralli, Annalisa Pace, Giampiero Gulotta, Stefano Pelucchi, Angelo Eplite, Claudio Vicini

**Affiliations:** 1 Department of Head-Neck Surgery, Otolaryngology, Head-Neck and Oral Surgery Unit, Morgagni Pierantoni Hospital, Via Carlo Forlanini, 34, 47121 Forlì, Italy; giannicola.iannella@uniroma1.it (G.I.); drmeccariello@gmail.com (G.M.); claudio@claudiovicini.com (C.V.); 2 Department of ‘Organi di Senso’, University “Sapienza”, Viale dell’Università, 33, 00185 Rome, Italy; giuseppe.magliulo@uniroma1.it (G.M.); ireneclaudia.visconti@gmail.com (I.C.V.); antonella.polimeni@uniroma1.it (A.P.); antonio.greco@uniroma1.it (A.G.); marco.devincentiis@uniroma1.it (M.d.V.); massimo.ralli@uniroma1.it (M.R.); annalisapace90@gmail.com (A.P.); giampierogulotta@gmail.com (G.G.); 3 Department of Cardiovascular, Respiratory, Nephrologic, Anaesthesiologic and Geriatric Sciences, Sapienza University, Viale dell’Università, 33, 00185 Rome, Italy; cristina.loiacono@uniroma1.it; 4 Laboratory of Anatomy and Cell Biology, Faculty of Medicine, University of Mons (UMONS), Avenue du Champ de Mars, 6, B7000 Mons, Belgium; jerome.lechien@umons.ac.be (J.R.L.); stefano.pelucchi@unife.it (S.P.); 5 Department ENT & Audiology, University of Ferrara, Via Savonarola, 9, 44121 Ferrara, Italy; prrtzn@unife.it; 6 Department of Medical and Surgical Sciences and Advanced Technologies “GF Ingrassia”, ENT Section, University of Catania, Via S. Sofia, 78, 95125 Catania, Italy; tnmaniaci29@gmail.com (A.M.); s.cocuzza@unict.it (S.C.); milenadilluca88@gmail.com (M.D.L.); 7 Department of Head-Neck Surgery, Otolaryngology, Head-Neck and Oral Surgery Unit, Ospedale “Santa Maria delle Croci”, Viale Vincenzo Randi, 5, 48121 Ravenna, Italy; dr.andrea.devito@gmail.com (A.D.V.); chiara.martone@hotmail.it (C.M.); 8 ENT Department, University of Milan, Via Festa del Perdono 7, 20122 Milan, Italy; angelo.eplite@unimi.it

**Keywords:** robotic surgery, CPAP, Obstructive Sleep Apnea, quality of life, sleep studies

## Abstract

Obstructive Sleep Apnea (OSA) syndrome is a respiratory sleep disorder characterized by a reduction (hypopnea) in or a complete cessation (apnea) of airflow in the upper airways at night, in the presence of breathing effort. The gold standard treatment for OSA is ventilation through continuous positive airway pressure (CPAP), although this often shows poor patient compliance. In recent years, transoral robotic surgery (TORS) has been proposed as a valid surgical treatment for patients suffering from OSA in a multilevel surgical setting. The aim of this study is to analyze the effects on QoL and daytime sleepiness of multilevel surgery for OSA (barbed pharyngoplasty + transoral robotic surgery). Furthermore, we compared the impact on QoL and daytime sleepiness of two different treatments for patients with moderate to severe OSA, such as CPAP and TORS. Sixty-seven OSA patients who underwent multilevel robotic surgery and sixty-seven OSA patients treated with CPAP were enrolled, defined as Group 1 and Group 2, respectively. The Glasgow Benefit Inventory (GBI) questionnaire was administrated to evaluate the changes in the QoL. Respiratory outcomes were evaluated and compared. Group 1 showed a GBI total average value of +30.4, whereas Group 2, a value of +33.2 (*p* = 0.4). General benefit score showed no difference between groups (*p* = 0.1). Better values of social status benefit (*p* = 0.0006) emerged in the CPAP Group, whereas greater physical status benefit (*p* = 0.04) was shown in the TORS Group. Delta-AHI (−23.7 ± 14.3 vs. −31.7 ± 15.6; *p* = 0.001) and Delta-ODI (−24.5 ± 9.5 vs. −29.4 ± 10.5; *p* = 0.001) showed better values in the CPAP group. Therapeutic success rate of the Multilevel TORS Group was 73.1% and 91% in the CPAP group (*p* = 0.01), respectively. Multilevel TORS and CPAP have a positive effect on the quality of life of OSA patients. Greater social support has been reported in the CPAP group and better physical health status in the TORS group. No statistical difference emerged in the reduction in daytime sleepiness between both groups.

## 1. Introduction

Obstructive Sleep Apnea (OSA) syndrome is a respiratory sleep disorder characterized by a reduction (hypopnea) in or complete cessation (apnea) of airflow in the upper airways at night, in the presence of breathing effort [1,2,3,4,5,6,7,8,9,10,11,12,13].

OSAS is a frequent and often underestimated disease, affecting between 2% and 4% of middle-aged women and men; the available literature suggests that OSA, in untreated adults, is associated with a poor health-related quality of life (HRQoL), probably due to symptoms and complications related to this disease [4,5].

Continuous Positive Air Pressure (CPAP) is considered the gold standard treatment for severe OSA and has been shown to be effective in reducing the apnea–hypopnea index (AHI), improving sleep quality and decreasing cardiovascular comorbidities [3,6]. In addition, it has been proven that the CPAP treatment is directly correlated to an improvement in the quality of life (QoL) of these patients [3,5,12,13,14], by restoring the sleep quality and OSA symptoms. Avlonitou et al. [14], who assessed the quality of life and symptoms of OSA patients after adhering to 6 months CPAP treatment, reported an improvement in the total quality of life index score (3.8 ± 0.9 vs. 5.8 ± 0.8, *p* < 0.01).

Over the years, different types of surgeries of the velo-pharyngeal region and/or on the tongue base, as an alternative to the ventilatory therapy, have been proposed (uvulopalatopharyngoplasty, barbed pharyngoplasty, expansion sphincter pharyngoplasty, maxillomandibular advancement, hyoid suspension, genioglossus advancement, etc.). Transoral Robotic Surgery (TORS) allows, in selected cases of OSA patients, the muscular and lymphatic resection of the tongue base, increasing the respiratory space and stabilizing the collapse of this anatomical region. TORS could also be combined with several types of pharyngoplasties in a multilevel surgical setting.

Surgical treatment in OSAS patients could have some important benefits, including the possible resolution of apneas/hypopneas, which reduce the early mortality and cardiovascular risk. Moreover, surgery could be considered a one-shot procedure, which allows patients to avoid the use of uncomfortable devices (CPAP and/or Mandibular advancement devices) every night of their life to treat OSA. Besides, most side effects of CPAP therapy (pressure effect of the mask, poor compliance, noise, etc.) could be avoided with surgery [3,4,5,6,7,8,9,10].

In the last years, many papers have confirmed the efficacy of TORS, performing it as a single-level surgery or as part of multilevel surgery [7,8,9,10,11].

In our clinical experience, more than 300 OSA patients treated with TORS in multilevel surgery showed an improvement in the post-operative quality of life, both for the reduction in OSA symptoms and also for the suspension of treatment with CPAP. However, to our knowledge, no studies in the literature have evaluated patients’ quality of life after multilevel surgery, including a transoral robotic approach for OSA treatment. Besides, no studies have compared changes in patients’ quality of life and the excessive daytime sleepiness of surgical and non-surgical OSA treatments. The aim of this study is to analyze effects on QoL of the multilevel surgery for OSA (barbed pharyngoplasty + transoral robotic surgery). Further, we have compared the impact on QoL of two different treatments for patients with moderate to severe OSA, such as CPAP and TORS.

## 2. Materials and Methods

### 2.1. Trial Design and Subjects of the Study

This is a retrospective bi-center study performed at the Otolaryngology, Head and Neck and Oral Surgery Department of Morgagni Pierantoni Hospital of Forlì and at the Otolaryngology Department of Sapienza University of Rome.

Subjects eligible for study inclusion were initially selected from patients with OSA treated in these centers with two different types of treatment: multilevel surgery for OSA (barbed pharyngoplasty + transoral robotic surgery + septoplasty) and a conventional treatment with CPAP.

Patients who underwent multilevel surgery were treated at the Otolaringology Department of the Morgagni Pierantoni Hospital of Forlì, from January 2017 to February 2019. These were exclusively patients with moderate–severe OSA refusing a CPAP treatment and showing a velo-pharynx and base of tongue collapse + nasal obstruction. Multilevel surgery consists of the resection of the tongue base using the Transoral Robotic Surgery (TORS) + epiglottoplasty + tonsillectomy + Barbed Repositioning Pharyngoplasty (BRP) + settoplasty and/or turbinoloplasty. This combined multilevel surgical approach is a standardized protocol, well described in the literature, that has been performed with good results for more than 10 years. Surgical steps were performed in all patients as reported in the original study of Vicini et al. [8,10,15].

In contrast, patients designated to CPAP therapy were moderate–severe OSA patients, not candidates for surgery (multiple collapses, poor oral opening, lateral hypopharyngeal collapse, fear of surgery), who well tolerated CPAP treatment.

Patients treated with these two different therapeutic approaches were subsequently screened in accordance with stringent inclusion and exclusion criteria to be enrolled in the two different study groups:

Patients enrolled in Group 1 are OSA patients treated with multilevel surgery. Inclusion criteria are: aged between 18 and 65 years old, moderate and severe OSA, at least 6 months of follow-up. Exclusion criteria are: patients lost to follow-up, smokers, patients treated with isolated TORS, patients with incomplete preoperative and postoperative data of the home sleep apnea study, patients who did not agree to perform the GBI questionnaire.

Patients enrolled in Group 2 are OSA patients treated with CPAP device. For inclusion in the study only patients with a mean age between 18 and 65 years, moderate–severe OSA patients and auto-CPAP device have been considered. Exclusion criteria are as follows: smokers, non-constant use of CPAP (less than 4 h/night), patients lost follow-up or with incomplete pre- and post-treatment data or patients who did not agree to perform the GBI questionnaire.

The number of patients in these groups was chosen in accordance with the sample size calculation. According to a margin of error of 5% and a confidence level of 95%, the population size required for each group was 67 patients. An identical number of patients were enrolled in the two groups to better compare their results.

### 2.2. Quality-of-Life Investigation

The Glasgow Benefit Inventory (GBI) [16,17] questionnaire was used to evaluate changes in the QoL of investigated patients [17]. It is a validated questionnaire able to measure the QoL changes related to a specific surgical or medical intervention. It is only designed for post-intervention use and is the most widely used method for evaluating the improvement of QoL in otorhinolaryngology and in other medical and surgical disciplines.

All patients considered eligible for enrolment in the study were contacted by two co-authors of the study and the validated Italian version of the Glasgow Quality of Life questionnaire was administered to each patient.

The questionnaire, which can be completed by interview or self-completed by patients, consists of 18 questions answered using a five-point Likert scale, addressing change in health status (post treatment for OSA).

The 18 questions of the GBI were evaluated, and a total score (Overall Benefit) was calculated. The total score can range from −100 (poorest outcome), through 0 (no change), to +100 (best outcome). It indicates the overall impact of the surgical or medical treatment on patients’ quality of life.

The Glasgow Benefit Inventory is further subdivided into three distinct subscales. Twelve questions focus on general changes in health status, as well as changes in psychosocial health status identifying the ‘General’ subscale. Moreover, three questions are related to the amount of social support needed in relation to the condition being questioned (Social). The remaining three questions address changes in physical health status including medication requirements and number of medical examinations required (Physical). As a result, in addition to the total score, it is possible to obtain a partial general score, a partial score on social support and one on physical health.

The mean time between surgery/CPAP activation and questionnaire administration was analyzed.

### 2.3. Evaluation of OSA Treatment Outcomes

The Epworth Sleepiness Scale (ESS) was used to assess daytime sleepiness. The ESS is a self-administered questionnaire with 8 questions. Respondents are asked to rate, on a 4-point scale (0–3), their usual chances of dozing off or falling asleep while engaged in eight different activities. Most people engage in those activities at least occasionally, although not necessarily every day. The ESS score (the sum of the 8-item scores, 0–3) can range from 0 to 24. The higher the ESS score, the higher that person’s average sleep propensity in daily life, or their ‘daytime sleepiness’ [10,11,12,13,14,15,16,17].

The home sleep apnea study (HSAT) was performed on each patient in both groups before either surgery or CPAP treatment were re-examined and all respiratory data collected. In the TORS group the post-treatment respiratory outcomes were collected from the HSAT performed at the last follow-up. In the CPAP group the respiratory outcomes were extracted from an HSAT performed simultaneously with the last control of the device (at least 6 months after CPAP activation).

In all cases the sleep studies were performed in an unattended manner by means of a Polymesam Unattended 8-channel Device. The following parameters were recorded during the sleep study: respiratory movement and airflow, heart rate, arterial oxygen saturation, position of patient and sleep time. The apnea–hypopnea index (AHI), Oxygen Desaturation Index (ODI), and the lowest SpO2 (LOS) were collected and recorded by experts in sleep medicine according to the American Academy of Sleep Medicine (AASM) guidelines [1,2,3,4,5,6,7,8,9,10,11,12,13,14,15,16,17,18].

Delta AHI (postoperative AHI minus preoperative AHI), Delta ODI (postoperative ODI minus preoperative ODI) and Delta LOS (postoperative LOS minus preoperative LOS) were calculated in order to express the value of surgical efficacy and to compare the two groups of patients. Therapeutic success was defined, according to the existing literature (Sher’s criteria), as the achievement of a postoperative value of AHI < 20 and a 50% improvement in the preoperative AHI value [18].

Patients with incomplete HSAT data or recent treated cases (post-treatment HSAT evaluation shorter than 6 months) were excluded from the study.

### 2.4. Statistical Analysis

The methods utilized to analyze the data and compare the two groups of patients were differentiates based on the type of data. For the categorical data the chi-square test was used, for the difference between preoperative and postoperative outcomes the Wilcoxon test was employed and for comparing baseline, follow-ups and delta values between the two groups with a normal distribution, the student *t*-test was chosen. *p*-values < 0.05 were considered statistically significant.

A post-hoc correction used Benjamini–Hochberg’s false discovery rate, at a q-value of 0.05. All analyses were performed using the STATA 12.1 software (Stata Corp., College Station, TX, USA).

### 2.5. Ethical Statement 

This research study was performed in accordance with the principles of the Declaration of Helsinki and approved by the local Ethics Committee.

## 3. Results

Sixty-seven patients were enrolled in both of the groups.

Baseline data concerning age, gender and number of comorbidities in both groups are shown in Table 1.

Patients in the two groups did not present any difference in the average values for age and sex and in the number and type of comorbidities present in each individual in each group (*p* > 0.05 for each class analyzed) (Table 1). As shown in Table 1, no statistical difference emerged in the pre-treatment respiratory data collected for both groups (*p* > 0.05 in each group comparison).

Besides, the groups studied showed similar time intervals from treatment and performance of the quality-of-life investigations. In Group 1, the mean average time from surgery to the QoL investigation was 3.4 years (range 2.5–4.8 years), whereas in Group 2, this period of time was estimated to be 3.2 years (range 2.9–4.2 years).

### 3.1. Quality of Life

In patients treated with TORS, a GBI total average value of +30.4 was obtained, with a maximum of +75.0 and a minimum value of −16.7. In the group of patients treated with CPAP, the total GBI average value was found to be +33.2, with a range between +88.9 and −11.1. No statistical difference emerged comparing the two study groups, regarding the total GBI value (*p* = 0.4) (Table 2).

General benefit, social benefit score and the physical score of both groups showed positive values; values are reported in Table 2. Comparing the two groups of patients, there was no statistical difference in the general benefit score (*p* = 0.1). Differently, the two groups of patients studied showed a statistical difference in the values of social status benefit (*p* = 0.0006), which appeared to be better in the CPAP group, and in the physical status benefit (*p* = 0.04), which, on the contrary, was greater in the TORS group. (Table 2).

### 3.2. OSA Treatment Outcomes

**Figure 1:**
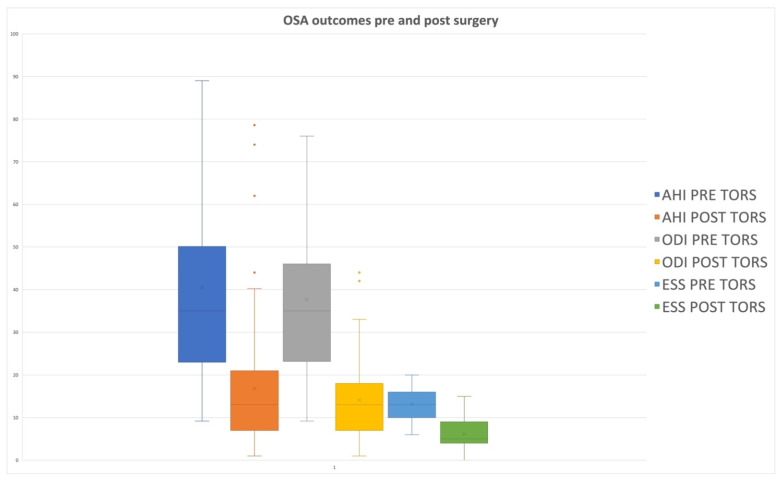
Box plot showing differences in OSA outcomes pre- and post TORS in a multilevel setting. Apnea Hypopnea Index (AHI); oxygen desaturation index (ODI); Epworth Sleepiness Scale (ESS).

In Group 1, patients presented a mean preoperative AHI value of 40.3 ± 15.7, which was reduced to a postoperative value of 16.7 ± 13.4, with a statistically significant difference (*p* = 0.0001) (Table 3 and Table 4; Figure 1).

**Figure 2:**
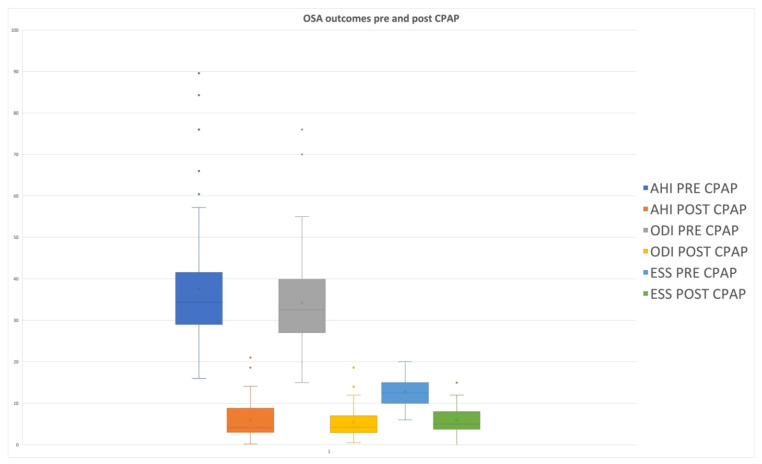
Box plot showing differences in OSA outcomes pre- and post-CPAP treatment. Apnea Hypopnea Index (AHI); oxygen desaturation index (ODI); Epworth Sleepiness Scale (ESS).

In Group 2, patients presented a mean preoperative AHI value of 37.7 ± 15.8 and a postoperative value of 6 ± 4.1, so that a statistically significant difference emerged between these preoperative and postoperative values (*p* = 0.0001) (Table 3; Figure 2). ODI and LOS data from the two groups of the study are reported in Table 3 and Figure 2. In both groups, a statistical difference between preoperative and postoperative average values of these parameters emerged.

ESS went down in Group 1, from 13.9 ±3.2 to 6.3 ±3.6 (*p* = 0.001), and in Group 2, from 12.8 ±2.8 5.8 ±2.5 (*p* = 0.001).

The Delta-AHI (−23.7 ± 14.3 vs. −31.7 ± 15.6; *p* = 0.001), as well as the Delta-ODI (−24.5 ± 9.5 vs. −29.4 ± 10.5; *p* = 0.001) of the two groups of patients revealed the best values in the CPAP group, with a statistically significant difference compared to the TORS group. Delta ESS was −7.6 ± 3.8 in Group 1 and −7 ± 3.9 in group 2. No statistical difference in daytime sleepiness reduction emerged between the groups.

The therapeutic success rate was found to be 73.1% in patients treated with multilevel surgery and 91% in patients treated with CPAP (*p* = 0.01) (Table 5).

## 4. Discussion

As only a few papers in the literature have investigated the improvement in quality of life after OSA treatments [19,20], limited data are available so far. Turner et al. [21] observed a statistically significant improvement (*p* < 0.04), in terms of working memory, long-term memory, quality of life and positive attitude after CPAP treatment. In their meta-analysis study, Kuhn et al. [19] proved, using the SF 36 questionnaire, a positive effect of CPAP treatment on the HR-QoL of patients with OSA.

Despite this published evidence, no studies in the literature have analyzed QoL after multilevel robotic surgery for OSA and no authors have compared multilevel surgery and CPAP therapy in the QoL improvement of OSA patients.

In this study, we analyzed the changes in QoL using the GBI questionnaire because it is a validated, standardized and widespread questionnaire that, performed after a specific surgical or medical intervention, is able to measure the QoL changes obtained. Moreover, it has the advantage of considering three sub-scales (general, social, physical), which makes it possible to investigate all aspects of the quality of life [16,17].

We analyzed the quality of life in patients surgically treated with multilevel surgery, including TORS, and in patients treated with CPAP. Comparable results were found in both groups of patients in the study; characteristics and severity of OSA in patients enrolled in the two groups were similar (see Table 1). Moreover, a similar period of time from the treatment and the quality-of-life investigations emerged for both groups.

In the surgically treated patients in our study, a GBI total average value of +30.4 emerged, with a maximum of +75.0 and a minimum value of −16.7. The general benefit score was calculated as +31.3 (range −63.8 to +88.3), and the social benefit was +11.5 (range 0 to +66.7), whereas the physical score was found to be +24.8 (range −16.7 to +50). These positive values over +20 points indicate an effective improvement in all aspects of the QoL.

Patients treated with CPAP also showed an excellent improvement in quality of life (+38.6 range −20.3 to +100). However, despite a higher mean value of the total GBI in this second group, no statistical differences emerged in the total GBI score (Overall Benefit) in comparison to surgically treated patients. These results could be explained by the good clinical and respiratory outcomes obtained by both the treatment methods analyzed [22]; a significant difference between pre-treatment and post-treatment AHI values (*p* = 0.0001) was observed in both groups of patients. Probably, for the same reason, a comparison of the two study groups did not reveal a statistically significant difference in the general partial score (*p* = 0.1). Similar results were described by Robinson et al. [23] in a clinical study: they did not observe a statistically significant difference between CPAP and Upper Airway Surgery (UAS), in terms of general quality of life (*p* = 0.308).

In terms of social subscale, we observed higher values in the CPAP group (mean +26.9) than in the TORS multilevel surgery group (mean +11.6), with a statistical difference (*p* = 0.0006). This subscale investigates the social support needed in terms of the help provided by relatives and friends in the management of daily life, in relation to the pathology and the proposed therapy. Probably, in CPAP patients, the fewer OSA symptoms (daytime sleepiness, headache, asthenia) are directly related to the improvement in their nocturnal respiratory values, which could explain the great benefit in their social support scale.

The physical score analyzes the benefits on physical health status, including frequency of illness, need of medicaments and medical consults. Regarding the scores of this subscale, statistical differences emerged between groups with higher values in the TORS group (mean 24.8) than in the CPAP group (mean 18.7 *p* < 0.04). In the case of surgical success [24,25], multilevel surgery could lead to a reduction in medical necessities and consults. Contrarily, patients who use CPAP require constant device setting and medical consults. Furthermore, this device is characterized by poor mask comfort, with a sense of pressure during the night. These aspects should not be underestimated, as they could be the cause of the poorer physical score observed in CPAP patients rather than in TORS subjects [26].

TORS in a multilevel setting and CPA did not show any difference regarding a reduction in daytime sleepiness, probably due to the positive effects of both treatment modes on nocturnal respiratory outcomes.

When comparing TORS and CPAP in QoL and respiratory outcomes, the adherence to CPAP therapy should be considered. It is well known that most patients use CPAP every night but do not use it for more than 4 h/night. Therefore, the overall effect of CPAP, even in ‘compliant’ patients, may be lower than in patients treated with multilevel surgery. Despite the fact that CPA may appear superior to TORS in AHI reduction and QoL improvement, low nocturnal adherence should also be considered. Results of surgery must be reviewed in this light, since surgery is an intervention that does not require the patient’s daily adherence once performed. Further studies are under way to evaluate the quality of life compared to the real use of CPAP.

Although important results regarding the impact of CPAP and TORS on quality of life and its various aspects have emerged, this study does present some limitations, such as its retrospective nature, the absence of a preoperative evaluation of QoL and the limited number of patients enrolled, risk of selection bias, lack of information regarding the baseline QoL information, HSAT instead of full polysomnography and, lastly, the retrospective nature of the study design. Moreover, surgically treated patients require a longer follow-up to confirm these statements. Further prospective studies on large series of patients are underway to validate these outcomes. Besides, differently to this preliminary study, it would be useful to analyze the differences in QoL between multiple subgroups of patients, including those with isolated velo-pharyngeal surgery and those with poor CPAP adherence. Another study limitation to consider is that all the QoL analyses in this study were based on questionnaires, which are very subjective and can be biased.

Finally, in our opinion, future studies should be performed to compare the quality of life between patients operated on with TORS versus other major surgical procedures for OSA, such as maxilla-mandibular advancement.

Maxillo-mandibular advancement (MMA) is an effective surgical option for the treatment of Obstructive Sleep Apnea (OSA), in patients who have difficulty tolerating continuous positive airway pressure and whose OSA is refractory to other surgical modalities. Maxillo-mandibular advancement achieves enlargement of the nasopharyngeal, retropalatal and hypopharyngeal airways by physically expanding the facial skeletal framework via Le Fort I maxillary and sagittal split mandibular osteotomies. This is a very effective approach that may, however, modify the physiognomy of the patients’ face and interfere with their post-surgical quality of life.

## 5. Conclusions

Multilevel surgery and CPAP treatment have a positive impact on the quality of life in OSA patients. Both therapies appear to be equally effective in the improvement in the general quality of life (total and general GBI scores), without statistical differences among them. Data show the need for greater social support in patients treated with CPAP, while those undergoing robotic multilevel surgery present a greater improvement in their physical health status.

## Figures and Tables

**Table 1:** Clinical characteristics and comorbidities of the two groups enrolled in the study. Abbreviations: Apnea Hypopnea Index (AHI); oxygen desaturation index (ODI); lowest SpO2 (LOS); Continuous positive airway pressure (CPAP); Transoral robotic surgery (TORS); Barbed reposition pharyngoplasty (BRP); Glasgow Benefit Inventory (GBI). Distribution of groups was normal and the outcomes have been compared using student *t*-test for continues variable whereas chi square test for categorical data; a value of *p* < 0.05 was considered statically significant.

	TORS + BRP + Septoplasty (Group 1) 67 Patients	CPAP (Group 2) 67 Patients	*p*-Value (Chi Square Test)
SEX			
Male	53	49	0.54
Female	14	18	
			***p*-Value** **(student *t*-test)**
Age	48.6 ± 6.5	51.2 ± 12.1	0.1
Number of comorbidities (arterial hypertension, cardiovascular problems, diabetes, cognitive problems)			
0	34 (50.7%)	31 (46.2%)	*p* > 0.05 (all cases)
1	20 (29.8%)	19 (28.3%)	
2	11 (16.4%)	14 (20.8%)	
>2	2 (2.9%)	3 (4.4%)	
Arterial hypertension	17 (25.3%)	19 (28.3%)	*p* > 0.05 (all cases)
cardiovascular problems (previous cardiac ischemic cardiopathy, rhythm disturbances, etc.)	5 (7.4%)	7 (10.4%)	
diabetes	4 (5.9%)	6 (8.9%)	
cognitive problems	5 (7.4%)	8 (11.9%)	
Pre AHI	40.3± 15.7	37.7 ± 15.8	*p* = 0.1
Pre ODI	37.8 ± 11.4	34.9 ± 13.9	*p* = 0.2
Pre LOS	80.6 ±6.9	81.4 ±6.6	*p* = 0.5
Pre ESS	13.9 ±3.2	12.8 ±2.8	*p* = 0.6
Pre BMI	29.3 ±4.2	30.8 ± 3.4	*p* = 0.1
Time between interventions and GBI questionnaire administration (years)	3.4 years (range 2.5–4.8 years)	3.2 years (range 2.9–4.2 years)	*p* > 0.5

**Table 2:** Glasgow Benefit Inventory (GBI) results of the two treatments for OSA investigated. Abbreviations: Continuous positive airway pressure (CPAP); Transoral robotic surgery (TORS); Barbed reposition pharyngoplasty (BRP); Glasgow Benefit Inventory (GBI).

	TORS + BRP + Septoplasty (Group 1) 67 Patients	CPAP (Group 2) 67 Patients	*p*-Value
Total score	Mean = +30.4 Standard Deviation = 24.2 Hi = +75.0 Low = −16.7	Mean = +33.2 Standard Deviation = 23.6 Hi = +88.9 Low = −11.1	0.4
General score	Mean = +31.3 Standard Deviation = 31.9 Hi = +83.3 Low = −63.9	Mean = +38.6 Standard Deviation = 26.5 Hi = +100. Low = −20.3	0.1
Social score	Mean = +11.5 Standard Deviation = 21.6 Hi = +66.7 Low = 0.00	Mean = +26.9 Standard Deviation = 28.0 Hi = +100. Low = 0.00	**0.0006**
Physical score	Mean = +24.8 Standard Deviation = 14.5 Hi = +50.0 Low = −16.7	Mean = +18.7 Standard Deviation = 29.1 Hi = +100. Low = −16.7	**0.04**

Distribution of groups was normal and the outcomes have been compared using student *t*-test; a value of *p* < 0.05 was considered statically significant.

**Table 3:** Statistical difference between pre- and post-treatment sleep apnea respiratory outcomes. Abbreviations: Apnea Hypopnea Index (AHI); oxygen desaturation index (ODI); lowest SpO2 (LOS); Continuous positive airway pressure (CPAP); Transoral robotic surgery (TORS); Barbed reposition pharyngoplasty (BRP); Glasgow Benefit Inventory (GBI). Changes from baseline within the groups were compared using the Wilcoxon test; a value of *p* < 0.05 was considered statically significant.

	Pre- Treatment AHI	Post- Treatment AHI	*p*-Value	Pre- Treatment ODI	Post- Treatment ODI	*p*-Value	Pre- Treatment LOS	Post- Treatment LOS	*p*-Value
Group 1	40.3 ± 15.7	16.7± 13.4	0.0001	37.8 ± 11.4	13.3 ± 10.4	0.0001	80.6 ±6.9	86.4 ±6.2	0.0001
Group 2	37.7 ± 15.8	6 ± 4.1	0.0001	34.9 ± 13.9	5.5 ± 5.8	0.0001	81.4 ±6.6	90.3 ± 5.3	0.0001

**Table 4:** Statistical difference between pre- and post-treatment sleep apnea respiratory outcomes. Body max index (BMI). Epworth Sleepiness Scale (ESS). Changes from baseline within the groups were compared using the Wilcoxon test; a value of *p* < 0.05 was considered statically significant.

	Pre- Treatment ESS	Post- Treatment ESS	*p*-Value	Pre- Treatment BMI	Post- Treatment BMI	*p*-Value
Group 1	13.9 ± 3.2	6.3 ± 3.6	0.0001	29.3 ± 4.2	27.9 ± 3.4	0.1
Group 2	12.8 ± 2.8	5.8 ± 2.5	0.0001	30.8 ± 3.4	28.5 ± 2.1	0.1

**Table 5:** Comparison between multilevel surgery and CPAP in post-treatment nocturnal respiratory outcomes evaluated using home sleep apnea testing (HSAT). Abbreviations: Apnea Hypopnea Index (AHI); oxygen desaturation index (ODI); lowest SpO2 (LOS); Continuous positive airway pressure (CPAP); Transoral robotic surgery (TORS); Barbed reposition pharyngoplasty (BRP).

	Group 1 TORS + BRP+ Septoplasty	Group 2 CPAP	*p*-Value
Delta-AHI	−23.7 ± 14.3	−31.7 ± 15.6	*p* = 0.001
Delta-ODI	−24.5 ± 9.5	−29.4 ±10.5	*p* = 0.001
Delta–LOS	−6.3 ±4.2	−9.7 ±3.9	*p* = 0.001
Delta–ESS	−7.6 ± 3.8	−7 ± 3.9	***p* > 0.5**
Success rate (AHI < 20 and 50% improvement in AHI) [19]	49/67 (73.1%)	61/67 (91%)	Chi-square test 0.01

Distribution of groups was normal and the outcomes were compared using student *t*-test; a value of *p* < 0.05 was considered statically significant.

## Data Availability

Not applicable.
